# Olfaction Contributes to Pelagic Navigation in a Coastal Shark

**DOI:** 10.1371/journal.pone.0143758

**Published:** 2016-01-06

**Authors:** Andrew P. Nosal, Yi Chao, John D. Farrara, Fei Chai, Philip A. Hastings

**Affiliations:** 1 Marine Biology Research Division, Scripps Institution of Oceanography, University of California San Diego, La Jolla, California, 92037, United States of America; 2 Department of Atmospheric and Oceanic Sciences and Joint Institute for Regional Earth System Science and Engineering, University of California Los Angeles, Los Angeles, California, 90095, United States of America; 3 School of Marine Sciences, University of Maine, Orono, Maine, 04469, United States of America; University of California Davis, UNITED STATES

## Abstract

How animals navigate the constantly moving and visually uniform pelagic realm, often along straight paths between distant sites, is an enduring mystery. The mechanisms enabling pelagic navigation in cartilaginous fishes are particularly understudied. We used shoreward navigation by leopard sharks (*Triakis semifasciata*) as a model system to test whether olfaction contributes to pelagic navigation. Leopard sharks were captured alongshore, transported 9 km offshore, released, and acoustically tracked for approximately 4 h each until the transmitter released. Eleven sharks were rendered anosmic (nares occluded with cotton wool soaked in petroleum jelly); fifteen were sham controls. Mean swimming depth was 28.7 m. On average, tracks of control sharks ended 62.6% closer to shore, following relatively straight paths that were significantly directed over spatial scales exceeding 1600 m. In contrast, tracks of anosmic sharks ended 37.2% closer to shore, following significantly more tortuous paths that approximated correlated random walks. These results held after swimming paths were adjusted for current drift. This is the first study to demonstrate experimentally that olfaction contributes to pelagic navigation in sharks, likely mediated by chemical gradients as has been hypothesized for birds. Given the similarities between the fluid three-dimensional chemical atmosphere and ocean, further research comparing swimming and flying animals may lead to a unifying paradigm explaining their extraordinary navigational abilities.

## Introduction

The mechanisms underlying pelagic underwater navigation have long captivated human observers, who, as instinctively visual navigators, would be utterly disoriented in this apparently uniform realm devoid of visible landmarks and beacons. Whereas terrestrial navigators may orient themselves using the position of the sun [1–2], for example, or the axis of celestial rotation [3–4], not even these cues are perceptible to underwater pelagic navigators except perhaps immediately below the surface. Furthermore, swimming movements in the open ocean are permanently uncoupled from stationary substrate, against which deflection due to current drift might otherwise be visually gauged and mitigated [5]. Thus, without non-visual means of compensation, the geographic path travelled may not reflect the intended movements of the animal swimming within constantly moving water parcels. These challenges notwithstanding, a variety of marine animals make routine oceanic migrations, including fishes, turtles, pinnipeds, and cetaceans, often between distant sites and along relatively straight paths [6]. Facilitating these migrations appear to be geomagnetic, chemical, and hydrodynamic cues, which are known to convey positional (map-sense) and directional (compass-sense) information to pelagic navigators; however, knowledge of how these cues are integrated over various spatial scales is underdeveloped and mostly limited to experiments involving sea turtles and salmonid fishes [7].

A more comprehensive understanding of the cues exploited by pelagic navigators requires a comparative approach, expanding to understudied taxa, including the cartilaginous fishes (Chondrichthyes), which have been shown to undertake directed movements through the open ocean [8]. For example, white sharks (*Carcharodon carcharias*) migrate between California and Hawaii, and to the conspicuous ‘offshore focal area’ or ‘café’ between the two [9]; scalloped hammerhead sharks (*Sphyrna lewini*) rhythmically disperse from islands and seamounts at night to forage in the surrounding pelagic environment [10]; salmon sharks (*Lamna ditropis*) travel between Alaska and the North Pacific Subtropical Gyre [11]; and tiger sharks (*Galeocerdo cuvier*) move between the islands of the Hawaiian Archipelago [12–13]. Among the potentially useful navigational cues, directed movements of sharks through the open ocean have mostly been attributed to geomagnetic orientation [10, 14–15], thought to be mediated indirectly via induction of the electrosensory system or directly via biogenic magnetite crystals [16–19]. However, multiple sensory modalities are likely integrated, as shown for other complex behaviours such as feeding [20], which together with spatial memory enables pelagic navigation and homing [21]. Other viable navigational cues may include sound, current, swell, wind, temperature, and, perhaps most interestingly, odorants.

Sharks are known for their keen sense of smell, but mostly as it relates to feeding [20, 22–25]. On the other hand, Jacobs [26] hypothesized that the primary function of olfaction is not the detection and discrimination of odours *per se*, but rather decoding and mapping odour distributions in space and time for the purpose of navigation. This ‘olfactory spatial’ hypothesis sought to explain why the vertebrate olfactory bulb (OB) does not scale allometrically with the rest of the brain [27–29] and predicts that OB scaling should reflect the adaptive value of tracking a dynamic chemical world and linking locations in olfactory space. Thus, OB size should scale with navigational demand independent of phylogeny. Yopak et al. [30] confirmed this prediction in sharks, demonstrating the largest OBs were found in migratory coastal-pelagic species such as the white shark (*C*. *carcharias*), tiger shark (*G*. *cuvier*), and blue shark (*Prionace glauca*). Although recent work by Gardiner et al. [31] has shown that olfaction participates in homing by juvenile blacktip sharks (*Carcharhinus limbatus*) within a shallow bay, the contribution of olfaction to open-ocean navigation in sharks has never been tested until now.

**Fig 1:**
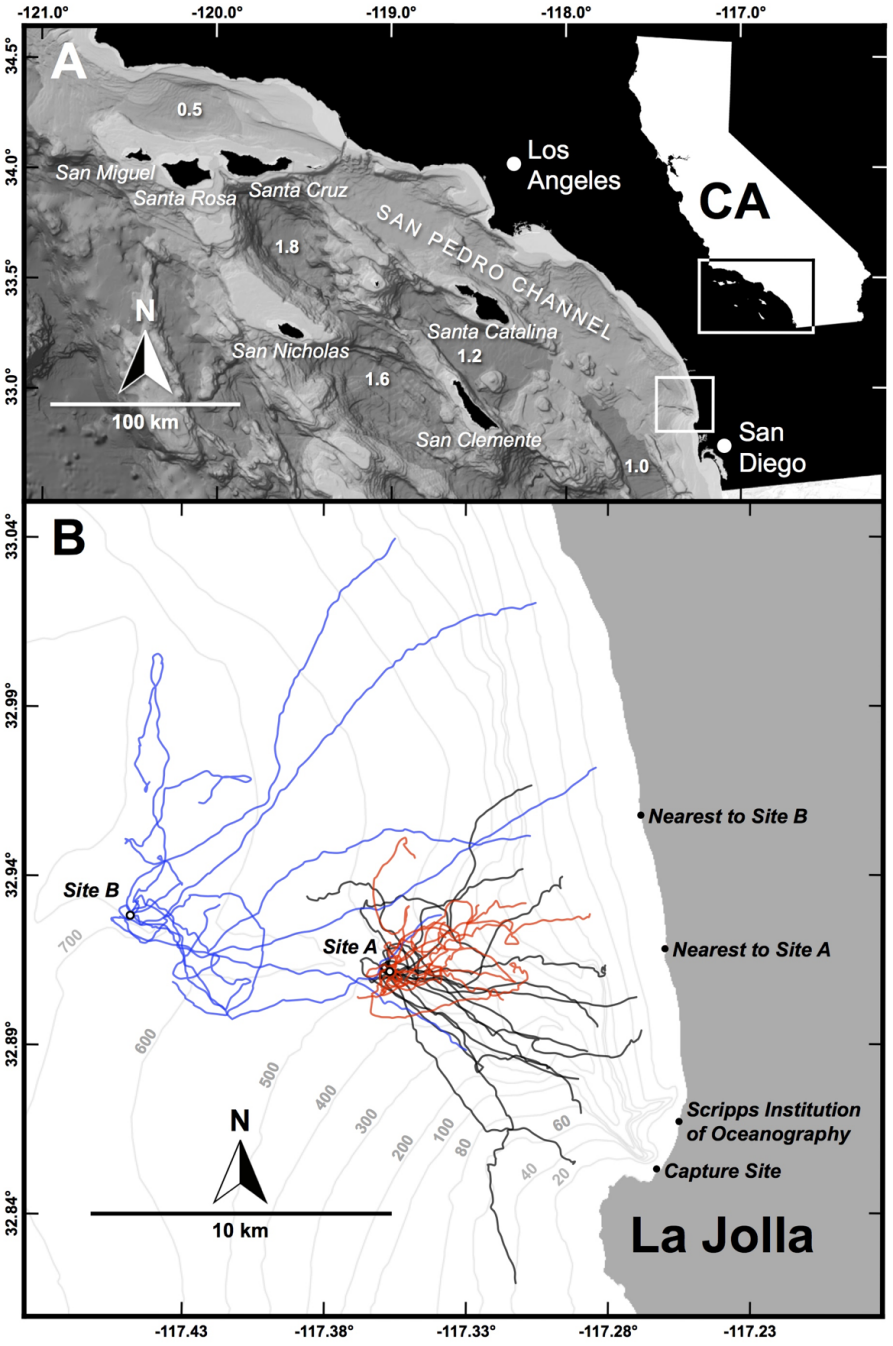
Swimming paths of experimentally displaced leopard sharks. A) Southern California Bight, zoomed in view of box in California (CA) inset map. The major Channel Islands are indicated in italics and various basin depths are indicated in km (bathymetry credit: NOAA). B) Zoomed in view of small box in A, showing the immediate study area. Bathymetry is shown at intervals of 20 m to 100 m, then at intervals of 100 m. Also shown are tracks (ground paths) of sharks released from Site A under anosmic (red) and sham (black) conditions and of sharks released from Site B under normal conditions (blue).

This study takes the first step toward understanding the contribution of olfaction to pelagic navigation in sharks, using the leopard shark (*Triakis semifasciata*) as a model. This typically nearshore species is endemic to the western coast of North America, where it forms seasonal aggregations in shallow, sheltered water, and feeds mostly on benthic invertebrates and fishes [32]. However, leopard sharks also make occasional forays into the pelagic environment, for example, crossing the San Pedro Channel between Santa Catalina Island and mainland California, which is 32 km wide at its narrowest point and approximately 800 m deep (Fig 1A) [33–34]. The mystery of how this otherwise nearshore benthic species navigates the open ocean was the impetus for this study, in which leopard sharks (some rendered anosmic) were transported from a nearshore aggregation site off La Jolla, California [34–35], up to 17 km offshore where bottom depths exceeded 500 m, released, and manually tracked using acoustic telemetry. It was hypothesized that these sharks would swim toward the nearest point on shore as their primary goal, so as to minimize their time in the hostile open ocean, which lacks their typical food and shelter from predators; the movements of anosmic sharks were hypothesized to be less efficient (e.g., slower, more tortuous) than sham-treated control sharks. Thus, the goals of this study were 1) to quantify the horizontal and vertical movements of leopard sharks during the pelagic phase of shoreward navigation and 2) to assess the importance of olfactory cues in pelagic navigation by comparing movements between anosmic and sham-control individuals.

## Methods

### Capture, Offshore Displacement, and Acoustic Tracking

The capture site (32.853°, -117.262°; bottom depth < 2 m; Fig 1B) was a known leopard shark aggregation that forms in June–December off La Jolla, California [34–35]. All sharks (*n* = 36) were captured from a 5-m skiff using handlines and baited barbless circle hooks, between 0720 and 0935 hrs in July–November of 2013–2014. Hooked sharks were guided into a large scoop net for hook removal. Then, netted sharks were transferred onto the deck of the skiff and turned ventral-side-up to induce tonic immobility. Eleven sharks were rendered anosmic by plugging the nares with cotton wool (0.5 g total per naris) soaked with petroleum jelly, using forceps. This two-minute minimally invasive procedure has been shown to block olfaction [20, 36–37] without the side effects that may accompany alternative methods of chemical ablation, numbing, or nerve severing. In fact, blacktip (*Carcharhinus limbatus*) and bonnethead sharks (*Sphyrna tiburo*) fed within several hours of this procedure [20]. Tests on captive leopard sharks indicated the plugs remained securely in place for at least 24 h, however, had begun to disintegrate. A sham version of this procedure was performed on another 15 leopard sharks, mimicking the handling and insertion of forceps, but without inserting cotton into the nares. Because real-time odorant sampling requires constant water flow across the olfactory lamellae, anything placed in the nares of sham-control sharks, even if resulting in only partial occlusion, would have obstructed this circulation and thus inhibited olfaction. This caveat notwithstanding, we describe below why any extraneous perturbation caused by the cotton plugs themselves would have likely been inconsequential. After the nare-plugging or sham procedure, netted sharks were released into a holding tank aboard the skiff (diameter = 1.0 m, depth = 0.6 m), containing 150 L seawater from the capture site.

En route to the release site, sharks were isolated from potentially retraceable cues. First, to mask geomagnetic cues, a strong neodymium ring magnet (grade N42; outer diameter = 2.54 cm, inner diameter = 0.64 cm) was suspended 5 cm above the centre of the bottom of the holding tank, such that it swung and spun randomly during transport. The strength of the magnetic field varied between 5 μT at 50 cm away and 535 μT at 10 cm away. Next, visual cues were occluded by covering the holding tank with an opaque tarp. To isolate the shark from chemical cues, no water changes were performed en route and the water was aerated only from a cylinder of compressed air filled at Scripps Institution of Oceanography (Fig 1B). Lastly, any potential inertial cues were masked by figure eight manoeuvres performed at the capture site just prior to departure and again just prior to arrival at the release site.

These sharks were transported 11.0 km NW to release Site A (32.911°, -117.357°; bottom depth = 550 m), 9.1 km from the nearest point on shore (Fig 1B); transit time was 1 hour. Upon arrival, the shark was measured, sexed, and tagged with a Floy Tag FIM-96 identification tag and a Vemco V16TP continuous acoustic transmitter with depth and temperature sensors, each via a nylon dart inserted into the musculature and through the radials on opposing sides of the base of the first dorsal fin. The V16TP transmitter was modified to have a syntactic foam float (Desert Star Systems) and galvanic timed release (model AA1, International Fishing Devices), allowing the transmitter to pop off, float to the surface, and be recovered and reused (S1 Fig). After tagging, sharks were released in a random direction and manually tracked using a Vemco VH110 directional hydrophone and VR100 acoustic receiver, until the transmitter apparatus resurfaced. GPS positions of the skiff, which by convention were taken to be the position of the shark, along with depth and temperature readings from the shark-borne transmitter, were recorded every 5 minutes. Lastly, the thermal structure of the water column was recorded using a Seabird SBE39 profiler, deployed every 30 minutes to a depth of 100 m or, if bottom depth was less than 100 m, to the bottom.

To provide additional context and a point of comparison to the above experiment, ten additional sharks were transported from the capture site 19.3 km WNW to release Site B (32.928, -117.448; bottom depth = 680 m), 17.2 km from the nearest point on shore (Fig 1B); transit time was 1.5 hours. These sharks were transferred to the holding tank immediately after hook removal, thus foregoing the tonic immobility and nare-plugging or sham procedure. For the purpose of discussion, these are termed ‘normal’ conditions, as opposed to the ‘anosmic’ or ‘sham-control’ conditions described above. A longer-duration model AA2 galvanic timed release was used to enable a longer tracking period. Otherwise, these sharks were treated the same as above. This study was approved by the Institutional Animal Care and Use Committee of the University of California—San Diego (Protocol S00080). Sampling and fieldwork were approved by the California Department of Fish and Game (Permit SC 9893).

### Data Analysis

Path tortuosity was measured using the fractal dimension (fractal *D*) [38–39] with modifications prescribed by Nams [40]. Fractal *D* is a continuous analogue of discrete geometric dimensions that ranges between 1 and 2, where 1 reflects a linear path (i.e., one-dimensional) and 2 a path so tortuous that it covers a plane (i.e., two-dimensional). The mean fractal estimator [40], implemented by the program FRACTAL v. 5.26 (V. Nams), was used to calculate the mean fractal *D* for each shark over a range of spatial scales (100–10,000 m). Briefly, gross distance was measured by walking 200 pairs of dividers of varying size along the path (i.e., 200 different spatial scales, equally spaced along the log-transformed span of 100–10,000 m), forward and backward, and averaging gross distance for each paired replicate. As divider size (spatial scale) increases, gross distance decreases, and the slope of the log-log plot of gross distance vs. spatial scale is 1 –*D*, with more tortuous paths yielding steeper slopes. Then, *D* was calculated for each spatial scale, centred within a narrow window spanning (spatial scale)/1.25 –(spatial scale)*1.25, which was slid along the x-axis from 100 to 10,000 m. The resulting *D* values were averaged to generate the mean fractal *D* for each shark, which was then transformed to log (*D*– 1) for statistical comparisons.

To determine whether sharks were performing directed (oriented) walks, we used FRACTAL v. 5.26 to implement the scaling test for oriented movement developed by Nams [41]. Briefly, an animal using a directed walk will make movement decisions that operate at large spatial scales, likely beyond the visual range. Animals performing unoriented movements will only make movement decisions that operate at small spatial scales (e.g. within visual range). Unoriented movements approximate a correlated random walk (CRW; the null model), which is a random walk but with directional bias over short time scales [41]. CRW_DIFF_, which is a measure of how movements compare to a CRW, was calculated at each spatial scale (using the same number and range of scales as the fractal analysis above). CRW_DIFF_ > 0 indicates greater displacement than a CRW, while CRW_DIFF_ < 0 suggests the movements are more constrained than a CRW [41]. An animal using a directed walk should thus yield CRW_DIFF_ > 0 at large spatial scales [8, 41].

A weighted mean vector (WMV; weighted by distance) was calculated for each shark using the program Oriana v. 4 (Kovach Computing Services). WMV bearings (degrees; north = 0° = 360°) and lengths (*r*, scaled 0–1; *r* = circular variance, *s*,– 1) were compared using Mardia Two-Sample and Mann-Whitney *U* Tests, respectively [42–43]. A grand WMV (weighted by WMV length) and 95% confidence interval were calculated for each condition according to Batschelet [42]. Lastly, the significance of each WMV and grand WMV was determined using Moore’s Modified Rayleigh Test, a non-parametric analogue of Rayleigh’s Test for weighted vector data [44]. To measure the fraction of forward swimming efforts directed toward shore, the hypothesized goal of the displaced sharks, a shoreward orientation index was calculated for each track as the ratio of mean shoreward speed to mean overall speed. Shoreward speed was calculated as [(distance to shore from point A, m)–(distance to shore from point B, m)] / (time interval between points A and B, s). Lastly, a simple measure of shoreward progress was calculated as [(distance to shore at start of track, m)–(distance to shore at end of track, m)] / (distance to shore at start of track, m), where a value of 1 indicates the shark reached the shoreline and negative values indicate the shark’s net movement was directed away from shore.

To assess the effect of current, these analyses were repeated on the reconstructed motor path of each shark, derived by subtracting current velocity (speed and direction) from each step of the tracked ground path. Current profiles were generated for every shark position (*n* = 2,212) with a vertical resolution of 1 m using the California coastal (CA) ocean forecasting system, based on the Regional Ocean Modelling System (ROMS). ROMS is a free-surface, hydrostatic, three-dimensional, primitive equation regional ocean model [45–47], configured here to consist of a single domain covering the entire California coastal ocean from north of Crescent City, CA to Ensenada, Mexico and extending approximately 1000 km offshore at a resolution of 3.3 km. In the vertical there are 40 unevenly spaced sigma levels with the majority of these clustered near the surface. Lateral boundary conditions were generated using output from a global HYCOM model (hycom.org) and surface atmospheric forcing is derived from hourly outputs from operational forecasts performed with the NCEP NAM 5-km North American model (http://www.emc.ncep.noaa.gov/index.php?branch=NAM).

A two-step multi-scale (MS) three-dimensional variational (3DVAR) data assimilation algorithm was used to generate the nowcast estimates of the three-dimensional ocean state. This MS-3DVAR scheme is a generalization of the 3DVAR methodology of Li et al. [48–49] and is described in detail by Li et al. [50]. The ROMS MS-3DVAR is designed to assimilate multiple types of observations simultaneously and reliably, while incorporating both the large-scale and small-scale impacts of the observations on the model fields, a distinct advantage over single-scale 3DVAR systems [51]. A number of surface and subsurface data were available in near real-time and assimilated by the ROMS MS-3DVAR system; a list of these is given in Farrara et al. [52]. An ecosystem model has been coupled with the ROMS MS-3DVAR system for the California Current System. The ecosystem model (CoSiNE—Carbon, Silicate, and Nitrogen Ecosystem) includes the following constituents: nitrate, silicate, ammonium, small phytoplankton, diatoms, micro- and meso-zooplankton, detrital nitrogen and silicon, total CO_2_ and alkalinity, and dissolved oxygen [53–54]. The coupled ROMS-CoSiNE produces hourly model outputs for all grid points. The chlorophyll estimates from the model were derived from the sum of small phytoplankton and diatoms with a constant conversion factor [54]. The modelled total biomass was the sum of small phytoplankton and diatoms, along with micro- and meso-zooplankton. Lastly, to demonstrate the potential for offshore and alongshore transport of dissolved odorants, ‘virtual drifters’ were released just off the coast at a depth of 20 m, directly east of Site A. For each simulation, 100 drifters were released and tracked for 17 days. This drifter release scenario was repeated every 15 days during the months of July–November of 2013–2014.

## Results

**Fig 2:**
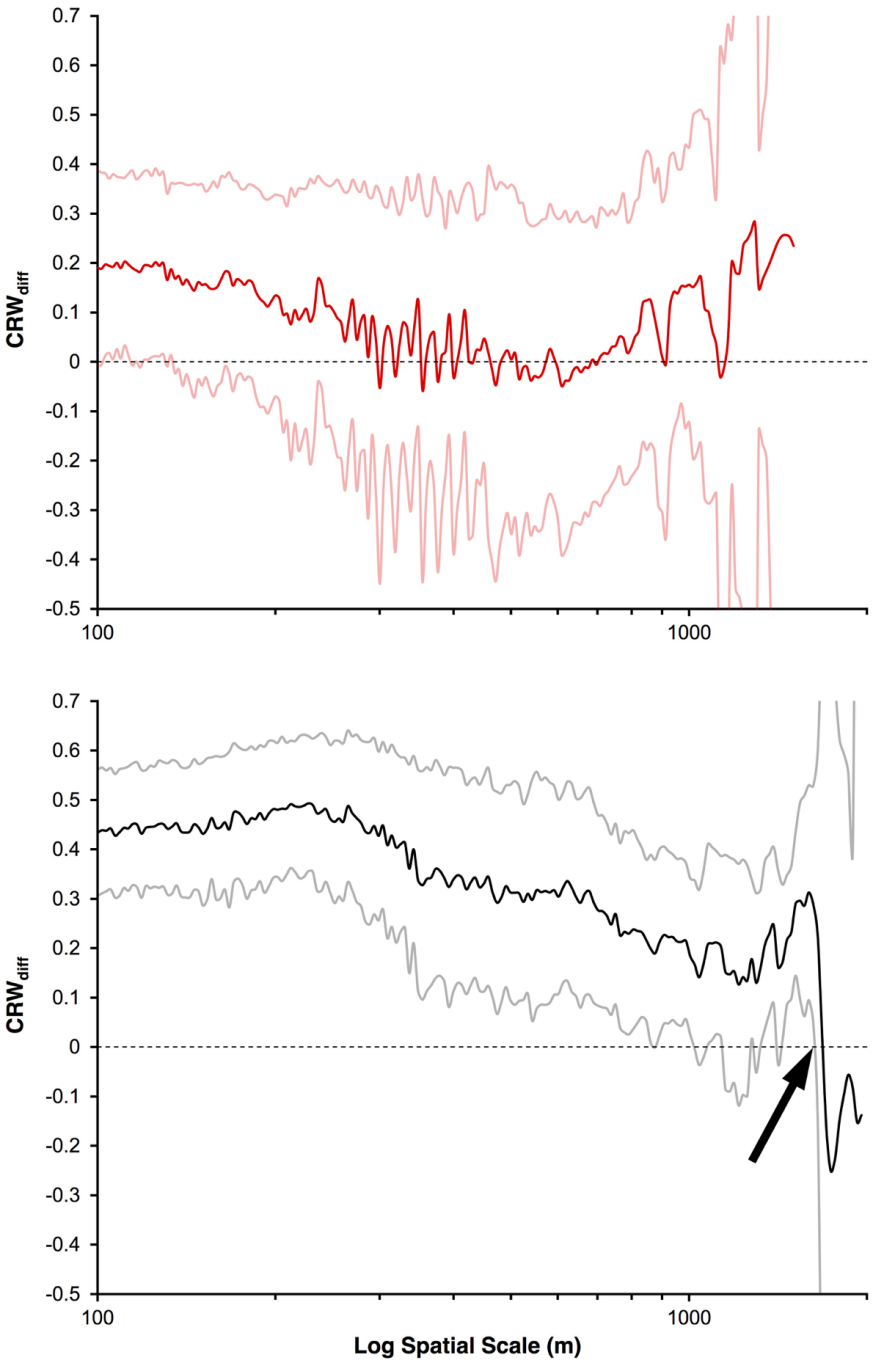
Comparison of leopard shark swimming paths to correlated random walk (CRW) using CRW_diff_ statistic. CRW_diff_ for anosmic (solid red line; top) and sham-treated sharks (solid black line; bottom) over various spatial scales with 95% confidence intervals (solid light red lines for anosmic and solid gray lines for sham-treated sharks). If CRW_diff_ > 0 (indicated by dashed black line), paths are oriented. If CRW_diff_ < 0, paths are unoriented. The black arrow indicates the largest scale (>1,600 m) of oriented movement in sham-treated sharks.

Sharks released from Site A were all mature females, with no significant difference in fork length, swimming depth, water temperature, or tracking period between anosmic and sham-control groups (Table 1). Release directions were uniformly distributed (i.e., no directional bias) in each of the three conditions (anosmic: Rayleigh’s *Z* = 0.58, Rao’s *U* = 141, *p* > 0.1; sham-control: *Z* = 1.25, *U* = 122, *p* > 0.1; normal: *Z* = 0.77, *U* = 135, *p* > 0.1), with no significant difference among the three conditions (Mardia-Watson-Wheeler *W* = 5.67, *p* = 0.23). Swimming depth generally coincided with the bottom of the thermocline and the subsurface maximum for chlorophyll a concentration (S2 Fig). At the time of transmitter release, which terminated the tracks, anosmic sharks had on average advanced 37% closer to shore, compared to 63% closer for sham-control sharks (Table 1; Fig 1B). Five sham-control sharks abruptly dove to the benthos after crossing over the continental shelf (Table 1; S3 Fig). Ground speed was significantly slower for anosmic sharks with a smaller fraction of this forward swimming effort directed toward shore (shoreward orientation index; Table 1). Path tortuosity, measured by fractal *D*, was also significantly higher for anosmic sharks than sham-control sharks (Table 1). Lastly, whereas sham-control sharks exhibited significantly oriented (directed) movements (CRW_diff_ > 0) over spatial scales exceeding 1600 m, anosmic sharks showed no oriented movements beyond the average step size (distance between successive GPS positions) of 150 m (i.e., movements approximated a correlated random walk at all spatial scales; Fig 2).

**Table 1:** Manual acoustic tracking results for experimentally displaced leopard sharks (*Triakis semifasciata*).

							Total	Mean	Mean	Weighted	Weighted			
			Fork	Track	Mean	Mean	Distance	Swimming	Swimming	Mean	Mean	Shoreward		
	Date		Length	Time	Depth	Temp	Traveled	Speed	Speed	Vector	Vector	Orientation	Shoreward	Mean
Condition	dd-mmm-yyyy	Sex	cm	h:mm	m	°C	km	ms^-1^	FLs^-1^	Bearing (°)	Length, *R*	Index	Progress	Fractal *D*
SITE A: ANOSMIC	10-Nov-2013	F	100	4:08	29.4	15.0	6.90 (7.13)	0.46 (0.48)	0.46 (0.48)	232 (206)	0.11 (0.16)	-0.17 (-0.15)	-0.17	1.38 (1.34)
SITE A: ANOSMIC	13-Nov-2013	F	124	4:13	28.9	14.4	8.46 (9.04)	0.56 (0.60)	0.45 (0.48)	71 (93)	0.23 (0.25)	0.49 (0.47)	0.46	1.19 (1.17)
SITE A: ANOSMIC	18-Nov-2013	F	125	4:13	35.2	14.6	6.62 (6.46)	0.44 (0.43)	0.35 (0.34)	53 (88)	0.29 (0.25)	0.49 (0.54)	0.36	1.17 (1.15)
SITE A: ANOSMIC	7-Jul-2014	F	134	4:09	26.4	16.0	6.00 (6.17)	0.40 (0.41)	0.30 (0.31)	49 (83)	0.22 (0.25)	0.34 (0.47)	0.23	1.24 (1.19)
SITE A: ANOSMIC	9-Jul-2014	F	121	4:01	17.9	22.2	6.87 (7.44)	0.48 (0.51)	0.40 (0.42)	103 (110)	0.26 (0.32)	0.53 (0.57)	0.40	1.14 (1.09)
SITE A: ANOSMIC	10-Jul-2014	F	121	3:57	26.6	19.8	5.31 (6.84)	0.37 (0.48)	0.31 (0.40)	94 (112)	0.44 (0.50)	0.78 (0.79)	0.46	1.05 (1.03)
SITE A: ANOSMIC	16-Jul-2014	F	110	3:59	13.4	17.2	7.06 (7.83)	0.49 (0.55)	0.45 (0.50)	90 (93)	0.34 (0.38)	0.59 (0.65)	0.47	1.15 (1.11)
SITE A: ANOSMIC	18-Jul-2014	F	127	4:21	13.1	17.3	7.61 (8.03)	0.49 (0.51)	0.39 (0.40)	63 (81)	0.32 (0.34)	0.64 (0.69)	0.53	1.14 (1.12)
SITE A: ANOSMIC	21-Jul-2014	F	131	4:27	16.1	15.4	8.92 (8.83)	0.56 (0.55)	0.43 (0.42)	12 (62)	0.16 (0.13)	0.18 (0.30)	0.17	1.25 (1.35)
SITE A: ANOSMIC	2-Oct-2014	F	132	5:26	33.0	16.0	10.8 (11.6)	0.55 (0.59)	0.42 (0.45)	74 (111)	0.38 (0.40)	0.64 (0.60)	0.75	1.10 (1.08)
SITE A: ANOSMIC	3-Oct-2014	F	131	5:02	35.9	16.1	10.0 (11.3)	0.55 (0.62)	0.42 (0.47)	94 (127)	0.19 (0.22)	0.41 (0.42)	0.45	1.23 (1.12)
		***SITE A*: *ANOSMIC MEANS*:**	**123**	**4:21**	**25.1**	**16.7**	7.69* (8.24*)	0.49* (0.52*)	0.40* (0.42*)	**76 (102)**	0.27* (0.29*)	0.45* (0.49*)	0.37*	1.17* (1.13*)
SITE A: SHAM	11-Oct-2013	F	123	3:42	24.4	14.5	8.28 (9.10)	0.62 (0.68)	0.50 (0.55)	111 (117)	0.57 (0.59)	0.74 (0.71)	0.68	1.04 (1.04)
SITE A: SHAM	12-Oct-2013	F	117	3:45	20.8	15.3	8.94 (8.59)	0.66 (0.64)	0.56 (0.55)	37 (48)	0.58 (0.55)	0.65 (0.75)	0.63	1.03 (1.03)
SITE A: SHAM	13-Oct-2013	F	127	4:07	25.6	14.6	9.65 (10.5)	0.65 (0.71)	0.51 (0.56)	95 (102)	0.49 (0.49)	0.83 (0.80)	0.88	1.04 (1.04)
SITE A: SHAM	14-Oct-2013	F	127	3:42	26.2	14.6	6.73 (6.51)	0.51 (0.49)	0.40 (0.39)	311 (118)	0.33 (0.49)	-0.34 (-0.34)	-0.25	1.15 (1.18)
SITE A: SHAM	19-Oct-2013	F	136	3:58	25.5	15.1	9.39 (10.0)	0.66 (0.70)	0.49 (0.51)	104 (111)	0.21 (0.22)	0.70 (0.69)	0.72	1.08 (1.06)
SITE A: SHAM	20-Oct-2013	F	135	3:56	25.4	15.1	8.88 (8.52)	0.63 (0.60)	0.47 (0.44)	61 (76)	0.52 (0.47)	0.70 (0.75)	0.69^+^	1.06 (1.06)
SITE A: SHAM	25-Oct-2013	F	130	3:54	26.0	14.6	9.84 (10.4)	0.70 (0.74)	0.54 (0.57)	119 (123)	0.52 (0.47)	0.59 (0.64)	0.64	1.10 (1.09)
SITE A: SHAM	6-Nov-2013	F	132	4:30	63.4	12.8	8.85 (10.4)	0.55 (0.64)	0.42 (0.48)	120 (140)	0.39 (0.45)	0.56 (0.62)	0.54	1.10 (1.06)
SITE A: SHAM	8-Jul-2014	F	127	4:30	35.0	18.8	7.88 (8.17)	0.49 (0.50)	0.39 (0.39)	53 (78)	0.39 (0.37)	0.64 (0.69)	0.56^+^	1.08 (1.08)
SITE A: SHAM	8-Aug-2014	F	132	5:18	16.3	17.3	11.4 (13.5)	0.60 (0.71)	0.45 (0.54)	135 (142)	0.33 (0.53)	0.69 (0.76)	0.87^+^	1.06 (1.03)
SITE A: SHAM	11-Aug-2014	F	131	5:54	12.2	20.3	13.2 (13.7)	0.62 (0.64)	0.47 (0.49)	157 (148)	0.43 (0.41)	0.48 (0.58)	0.69	1.03 (1.02)
SITE A: SHAM	21-Aug-2014	F	127	4:08	11.0	20.3	8.28 (9.27)	0.56 (0.62)	0.44 (0.49)	81 (94)	0.22 (0.28)	0.39 (0.50)	0.35	1.19 (1.13)
SITE A: SHAM	22-Sep-2014	F	123	5:35	47.3	14.8	10.1 (12.5)	0.50 (0.62)	0.41 (0.50)	125 (137)	0.33 (0.40)	0.68 (0.65)	0.74	1.07 (1.04)
SITE A: SHAM	25-Sep-2014	F	128	4:52	24.5	15.9	10.7 (11.5)	0.61 (0.66)	0.48 (0.52)	102 (104)	0.37 (0.39)	0.75 (0.77)	0.87^+^	1.03 (1.03)
SITE A: SHAM	13-Oct-2014	F	132	5:20	86.3	14.6	9.62 (12.3)	0.50 (0.64)	0.38 (0.48)	88 (118)	0.39 (0.49)	0.76 (0.70)	0.80^+^	1.07 (1.03)
		***SITE A*: *SHAM MEANS*:**	**128**	**4:28**	**31.3**	**15.9**	9.45* (10.3*)	0.59* (0.64*)	0.46* (0.50*)	**100 (110)**	0.40* (0.44*)	0.59* (0.62*)	0.63*	1.06* (1.05*)
SITE B: NORMAL	2-Jul-2013	F	131	6:48	16.2	15.4	15.3 (15.7)	0.63 (0.64)	0.48 (0.49)	4 (3)	0.18 (0.23)	0.09 (0.11)	0.08	1.27 (1.23)
SITE B: NORMAL	5-Jul-2013	F	132	6:39	8.2	19.0	16.3 (16.9)	0.68 (0.71)	0.52 (0.54)	78 (74)	0.43 (0.43)	0.81 (0.80)	0.77	1.04 (1.04)
SITE B: NORMAL	24-Jul-2013	F	138	6:29	15.8	16.7	14.2 (13.8)	0.61 (0.59)	0.44 (0.43)	112 (105)	0.26 (0.26)	0.76 (0.78)	0.62	1.04 (1.04)
SITE B: NORMAL	29-Jul-2013	F	134	6:30	1.9	18.9	13.3 (13.9)	0.57 (0.60)	0.43 (0.45)	31 (357)	0.14 (0.21)	0.21 (0.06)	0.17	1.31 (1.23)
SITE B: NORMAL	30-Jul-2013	F	128	6:37	5.9	17.1	18.9 (18.6)	0.79 (0.78)	0.62 (0.61)	72 (67)	0.50 (0.50)	0.83 (0.84)	0.91	1.03 (1.03)
SITE B: NORMAL	1-Aug-2013	F	126	7:03	16.3	13.4	16.4 (16.3)	0.65 (0.64)	0.52 (0.51)	34 (37)	0.59 (0.58)	0.72 (0.73)	0.69	1.02 (1.02)
SITE B: NORMAL	6-Aug-2013	F	131	6:45	16.7	15.4	18.1 (18.8)	0.75 (0.77)	0.57 (0.59)	52 (55)	0.41 (0.40)	0.84 (0.82)	0.89^+^	1.01 (1.01)
SITE B: NORMAL	14-Aug-2013	F	135	6:37	19.0	15.9	12.5 (12.6)	0.53 (0.53)	0.39 (0.39)	148 (112)	0.09 (0.04)	0.10 (0.09)	0.07	1.18 (1.19)
SITE B: NORMAL	20-Sep-2013	F	114	7:14	27.6	15.1	13.5 (13.1)	0.52 (0.50)	0.46 (0.44)	90 (89)	0.36 (0.37)	0.72 (0.72)	0.57	1.04 (1.05)
SITE B: NORMAL	30-Sep-2013	F	123	6:30	21.5	15.5	14.3 (14.3)	0.61 (0.61)	0.50 (0.50)	84 (56)	0.12 (0.13)	0.20 (0.17)	0.17	1.30 (1.22)
		***SITE B*: *NORMAL MEANS*:**	**129**	**6:43**	**14.9**^**S**^	**16.2**	**15.3 (15.4)**	**0.63**^**A**^ **(0.64**^**A**^**)**	**0.49**^**A**^ **(0.50**^**A**^**)**	**65 (58**^**AS**^**)**	**0.31 (0.32)**	**0.53 (0.51)**	**0.49**	**1.07 (1.06)**

Leopard sharks were released from Site A under anosmic (‘SITE A: ANOSMIC’; *n* = 11) and sham (‘SITE A: SHAM’; *n* = 15) conditions, and at Site B under normal conditions (‘SITE B: NORMAL’; *n* = 10) (Fig 1). Analyses of reconstructed motor paths (current-corrected) are shown in parenthesis immediately following the analyses of the tracked ground paths.

* indicates a significant (*p* < 0.05) difference between ‘SITE A: ANOSMIC’ and ‘SITE A: SHAM’ conditions.

A Mann-Whitney *U* test was used for each comparison, except Weighted Mean Vector Bearing, which was compared using a Mardia Two-Sample *U* test, Mean Fractal *D*, which was transformed to log (*D–* 1) and compared using Student’s *T* test, and Swimming Speed, which was also compared using Student’s *T* test.

^+^ indicates the shark ‘touched down’ on solid substrate after crossing over the continental shelf.

^A^ and ^S^ indicate a significant difference between ‘SITE B: NORMAL’ and ‘SITE A: ANOSMIC’ conditions and between ‘SITE B: NORMAL’ and ‘SITE A: SHAM’ conditions, respectively (Bonferroni-adjusted *p* < 0.05). The statistical tests are the same as above. Because Site B was purposefully farther from shore and track time was purposefully longer, Total Distance Travelled and Track Time were not compared.

Sharks released from Site A generally experienced an undercurrent flowing north and west, resulting in divergent reconstructed motor paths (S4 Fig). Subtracting current from the tracked ground path yielded the true motor speed, which, for anosmic sharks (0.42 FLs^-1^) approximated the theoretical optimal cruising speed (0.47 FLs^-1^; *U* = 84, *p* = 0.13) predicted by Weihs’ [55] equation, log (optimal cruising speed, ms^-1^) = 0.44 log (fork length, m)– 0.28, with modifications prescribed by Ryan et al. [56]. However, the motor speed of sham-control sharks (0.50 FLs^-1^) was significantly higher than the theoretical optimum (0.46 FLs^-1^; *U* = 178, *p* = 0.007). Correcting for current changed the grand weighted mean vector bearing from 76.1° to 102.0° for anosmic sharks (Moore’s Paired *R* = 1.732, *p* < 0.001) and from 99.9° to 109.8° for sham-treated sharks (Moore’s Paired *R* = 1.312, *p* < 0.01; S5 Fig). However, the current correction did not alter the results of fractal analysis or shoreward orientation index (Table 1).

Sharks released from Site B under normal conditions exhibited mixed results in terms of shoreward navigation ability. Four of these ten sharks appeared lost, having advanced only 7–17% closer to shore before their transmitters released, and exhibiting high fractal *D* (range: 1.18–1.31) and low shoreward orientation indexes (range: 0.09–0.21). By comparison, the other six sharks advanced 62–91% closer to shore before their transmitters released, with low fractal *D* (range: 1.01–1.04) and high shoreward orientation indexes (range: 0.72–0.84). These results did not change after current correction (Table 1). The motor speed of the ostensibly lost sharks (0.46 FLs^-1^) approximated the theoretical optimum (0.45 FLs^-1^), while that of the apparently oriented sharks (0.52 FLs^-1^) was higher than the optimum (0.46 FLs^-1^); however, due to small sample size, significance could not be tested. Nevertheless, the movements of the ostensibly lost sharks released from Site B resembled those of anosmic sharks released from Site A, while the movements of the apparently oriented sharks released from Site B resembled those of sham-control sharks released from Site A (Table 1).

## Discussion

Relatively little consideration has been given to chemical cues guiding animals through the pelagic environment, even though this dynamic three-dimensional medium in many ways resembles the dynamic three-dimensional atmosphere, where chemosensory modalities are widely accepted to participate in bird [57–60] and insect navigation [61–62]. Evidence for olfaction-mediated homing and navigation in fishes had heretofore been limited to salmonid fishes [63], rockfishes [64], and fish larvae [65–66], operating mostly in nearshore environments. Even the most recent work, which demonstrated olfaction-mediated homing in juvenile sharks, was conducted wholly within a shallow bay [31]. Although olfaction has also been hypothesized to contribute to pelagic navigation in sharks [8, 15, 67], this had never been tested until now.

This study is the first to demonstrate experimentally that olfaction participates in open-ocean navigation by sharks. Whereas sham-control leopard sharks released from Site A swam remarkably straight paths back to shore at speeds exceeding the theoretical optimal cruising speed (consistent with a degree of confidence as to the distance and direction to shore; Fig 1, Table 1), anosmic sharks swam at the most fuel-efficient speed along more tortuous routes that, as a group, did not exhibit directed movement. In contrast, control sharks exhibited directed movements over spatial scales exceeding 1600 m (Fig 2), which is comparable to adult thresher sharks (*Alopias vulpinus*) [8]. Given the substantial and identical physical discomfort and stress sustained by both anosmic and sham-control sharks (i.e., capture, transport in a dark and turbulent holding tank, tagging, and discharge in unfamiliar and hostile territory), any additional perturbation caused by the cotton plugs themselves was likely inconsequential. Moreover, the presence of predators and lack of suitable food should have fomented a strong and steadfast motivation to return efficiently to shore, a drive that was unlikely to have been quashed by the plugs themselves. Lastly, because disoriented behaviour was also observed in a few sharks whose nares were not plugged (four normal sharks and one sham-control shark; Fig 1, Table 1), the disoriented behaviour of anosmic sharks cannot be dismissed solely as an artefact of the plugs themselves. Rather, we contend that the disoriented behaviour of anosmic sharks was indeed caused by the inability to detect and behaviourally respond to chemical cues.

Cross-shore chemical gradients associated with coastal productivity could explain these observations. For example, the cross-shore chlorophyll a gradient is a relatively stable feature off California, where the concentration of chlorophyll a increases with proximity to shore due to coastal upwelling. The sharks are not likely smelling the pigment *per se*, but rather other biological compounds correlated with productivity. For example, the sharks could be detecting dissolved free amino acids, which are found in plankton exudates. Amino acids are hypothesized to be the primary odorant guiding salmon to their natal streams [68] and sharks are known to be able to detect amino acids down to nanomolar concentrations, which is comparable to teleost fishes, and strong enough to detect ambient levels of amino acids in the marine environment [25]. Another possible chemical cue is dimethyl sulphide (DMS), a fragrant compound produced by degrading dimethylsulfoniopropionate (DMSP), a common metabolite of phytoplankton and other marine algae [69]. Biogenic DMS has been hypothesized to function as a navigational and foraging cue for seabirds [58, 70], marine mammals [71], and even planktivorous sharks [72–73]. Recent work by Dove [74] confirms that whale sharks (*Rhincodon typus*) detect and behaviourally respond to DMS dissolved in seawater. Interestingly, the swimming depths of the tracked leopard sharks generally coincided with the subsurface chlorophyll a and biomass maxima, relatively stable features that historically have co-occurred at a mean depth of 21 m with a thickness of 13 m in the vicinity of our experiment [75]. The nowcast chlorophyll a and biomass profiles modelled in this study were very similar (S2 Fig). More important is that near the subsurface maxima is where the greatest horizontal variation occurred, yielding the steepest cross-shore gradients. Although the ability of sharks to discriminate different concentrations of chemical cues is not known, swimming at these depths, where gradients are steepest, would likely facilitate discrimination.

Whereas simple cross-shore chemical gradients provide information in one dimension, generally perpendicular to shore, a more complex ‘odorscape’ with multiple intersecting gradients would be necessary for two-dimensional chemical navigation. These could be combinations of biogenic and abiotic odorants emanating from eroding rocks, river outflows, reefs, or kelp forests, such that geographic locations have unique chemical signatures, akin to those identifying natal streams to homing salmon. Using virtual drifters, we demonstrated strong potential for offshore and alongshore transport of coastal odorants (S6 Fig) and recent work by Combes et al. [76] confirmed that stable gradients do indeed form by continuous dispersal of passive tracers via currents and advection. Similarly, Wallraff [60] confirmed that gradients of atmospheric trace gases yield sufficient spatial information for navigation by birds over hundreds of kilometres. Although the scales over which olfaction-mediated navigation operate in the pelagic environment are unknown, scales of at least tens of kilometres seem likely. Approximately half of the sharks released from Site B, 17 km from shore, found their way back to shore, while the other half were ostensibly lost (Fig 1). This could be explained by chemical gradients being weaker or patchier with distance from shore or else the mechanism and environmental cues used are different and only the most experienced sharks are able to navigate.

Although the paths of anosmic leopard sharks approximated a correlated random walk, these movements were nonetheless biased toward shore (Table 1, Fig 1B, S5 Fig) and may very well have reached the shore some time after their transmitters released. This indicates some knowledge of the location of shore was provided by non-olfactory cues. These could still be chemical, but mediated by taste instead of smell; however, the scale over which gustation operates is likely much smaller. Horizontal temperature gradients could also be useful (e.g., temperature generally decreases with proximity to shore due to coastal upwelling); however, their detection would be confounded by much stronger vertical gradients. The time-compensated position of the sun could provide directional information, as it does for birds [1–2]. However, several leopard sharks appeared lost even on completely sunny days, including the shark released on 29-Jul-2013, which had the shallowest mean swimming depth of any shark (1.9 m; Table 1) and thus the greatest chance of perceiving the position of the sun underwater. Meanwhile, other sharks successfully navigated to shore on completely overcast and foggy days. Although we cannot rule out orientation to polarized light, the biophysical mechanism in fishes relies on ultraviolet-sensitive cones [77]; these have not been identified in sharks, which exhibit cone monochromacy with wavelengths of maximum absorbance outside the ultraviolet spectrum [78].

In contrast, ambient noise could be a very useful navigational cue that has been suggested to participate in shark homing [15, 67]. For example, low-frequency surf noise could indicate the location of shore and, off La Jolla, California, surf noise is loudest at approximately 50–300 Hz [79], which is well within the hearing range of sharks [22]. Furthermore, the leopard sharks tracked in this study mostly swam near the bottom of the thermocline, where sound travels slowest and farthest (S2 Fig), thus facilitating the detection of sounds emanating from shore. Another interesting observation was that shortly after crossing back over the continental shelf, some sharks, even after swimming for hours at relatively constant depths, suddenly and deliberately dove to the benthos, as if they were confident a bottom of suitable depth was there (S3 Fig). Surely the sharks could not see the bottom from 50 m above it, but the ‘soundscape’ may be fundamentally different over the shallow shelf compared to deeper offshore areas. Lastly, geomagnetic cues are strongly suspected to play a role in shark navigation [10, 14–15] and these may very well contribute to shoreward navigation by leopard sharks. In short, olfaction plays a role in pelagic navigation, but is apparently supplemented by other sensory modalities, warranting further work to elucidate how these are integrated and organized hierarchically for navigation.

Evidence supporting Jacobs’ [26] ‘olfactory spatial’ hypothesis is growing in numerous vertebrate taxa, including the cartilaginous fishes [30], and identifying a unifying paradigm explaining the extraordinary navigational abilities of phylogenetically unrelated animals, particularly those that fly through the air and those that swim through the water, seems inevitable. Animals navigating in either medium face similar challenges, and given the growing body of evidence supporting olfaction-mediated navigation by flying birds and insects [57–62], along with the results of this study, olfaction-mediated navigation by underwater navigators is likely common, if not universal.

## Supporting Information

S1 Fig Reusable tagging apparatus, showing the modified Vemco V16TP transmitter with a galvanic timed release (GTR) and syntactic foam float (spray-painted orange). A Floy Tag FIM-96 identification tag was glued to the syntactic foam float to facilitate sighting and recovery at the surface (right photo; tagging apparatus having detached from shark, shown by white arrow).
(JPG)

S2 Fig Mean swimming depths of anosmic (released from Site A; dashed red line), sham-control (released from Site A; dashed black line), and normal leopard sharks (released from Site B; dashed black line). Also shown is mean thermal profile along tracks of sharks released from Site A (solid black line) and Site B (solid blue line), as well as mean chlorophyll a profile along tracks of all sharks (solid dark green line with mean ± SD indicated by solid light green lines).
(PNG)

S3 Fig Final approaches to shore of two representative leopard sharks with “touch-down” onto the benthos after crossing over the continental shelf. Depth data (black dots connected by solid black lines) are shown at 1-min resolution, spanning a window of 60 minutes. Bottom depth is indicated by thick gray line. The sham-treated shark tracked on 8-Jul-2014 is shown on the left with her abrupt dive to the bottom commencing at 4 h 15 min after release from Site A. The shark tracked on 6-Aug-2013 under normal conditions is shown on the right with her abrupt dive to the bottom commencing at 6 h 17 min after release from Site B.
(PNG)

S4 Fig Effect of current on leopard shark movements released from Site A. Mean current profiles over tracks of sharks released from Site A are shown on the left graph, with the zonal component as a solid black line (negative is west and positive is east) and the meridional component as a dashed black line (negative is south and positive is north). To the right are representative tracked ground paths and reconstructed motor paths of sharks released from Site A. Tracked ground paths are indicated by solid black (sham-treated) and red (anosmic) lines, while the reconstructed motor paths are indicated by gray (sham-treated) and light red (anosmic) lines. The four sham-treated examples are shown from the same release point because they could be easily combined without overlap. The four anosmic examples are separated for clarity, but the release point is Site A in all cases.
(PNG)

S5 Fig Mean weighted vector (MWV) bearings and lengths (*r*) for tracked ground paths of sham-treated (black arrows, *n* = 15) and anosmic (red arrows; *n* = 11) leopard sharks released from Site A and normal sharks released from Site B (blue arrows, *n* = 10). The bold arrow in each plot represents the grand MWV for the group. Shaded wedges in each plot represent the 95% confidence interval for the grand MWV.
(JPG)

S6 Fig Zoomed in view of box in California (CA) inset map. The extent of offshore and alongshore transport shown by small black dots, which indicate the simulated tracks of clusters of 100 virtual drifters released just offshore at a depth of 20 m (black arrow), directly east of release site A. Release site B is shown for reference. These drifters were released every 15 days during July–November of 2013–2014 and tracked for 17 days.
(JPG)
