# Supplementary figures and images for: Olfaction Contributes to Pelagic Navigation in a Coastal Shark
Source: PLoS One. 2016 Jan 6;11(1):e0143758. doi: 10.1371/journal.pone.0143758 (PMC4703295; doi:10.1371/journal.pone.0143758)

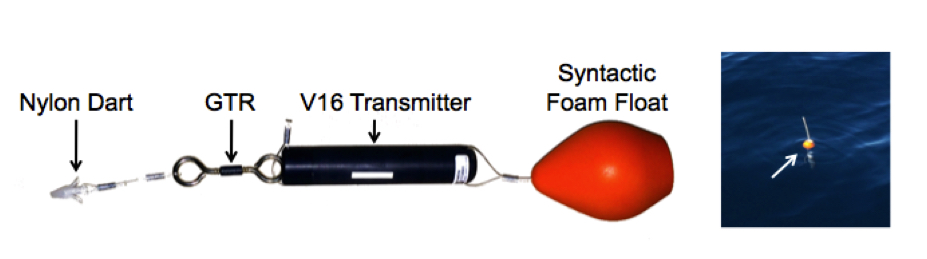

Supplement: S1 Fig — A Floy Tag FIM-96 identification tag was glued to the syntactic foam float to facilitate sighting and recovery at the surface (right photo; tagging apparatus having detached from shark, shown by white arrow). (JPG) [file pone.0143758.s001.jpg]

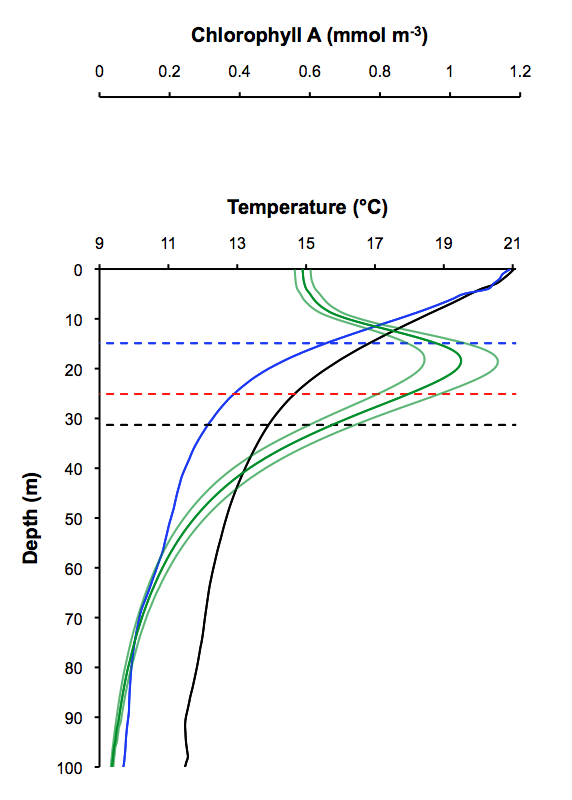

Supplement: S2 Fig — Also shown is mean thermal profile along tracks of sharks released from Site A (solid black line) and Site B (solid blue line), as well as mean chlorophyll a profile along tracks of all sharks (solid dark green line with mean ± SD indicated by solid light green lines). (PNG) [file pone.0143758.s002.png]

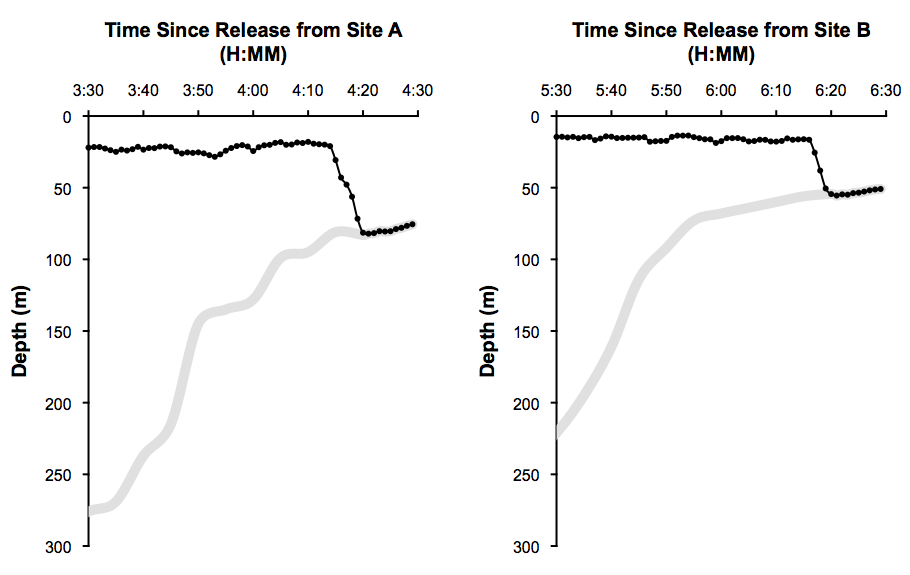

Supplement: S3 Fig — Depth data (black dots connected by solid black lines) are shown at 1-min resolution, spanning a window of 60 minutes. Bottom depth is indicated by thick gray line. The sham-treated shark tracked on 8-Jul-2014 is shown on the left with her abrupt dive to the bottom commencing at 4 h 15 min after release from Site A. The shark tracked on 6-Aug-2013 under normal conditions is shown on the right with her abrupt dive to the bottom commencing at 6 h 17 min after release from Site B. (PNG) [file pone.0143758.s003.png]

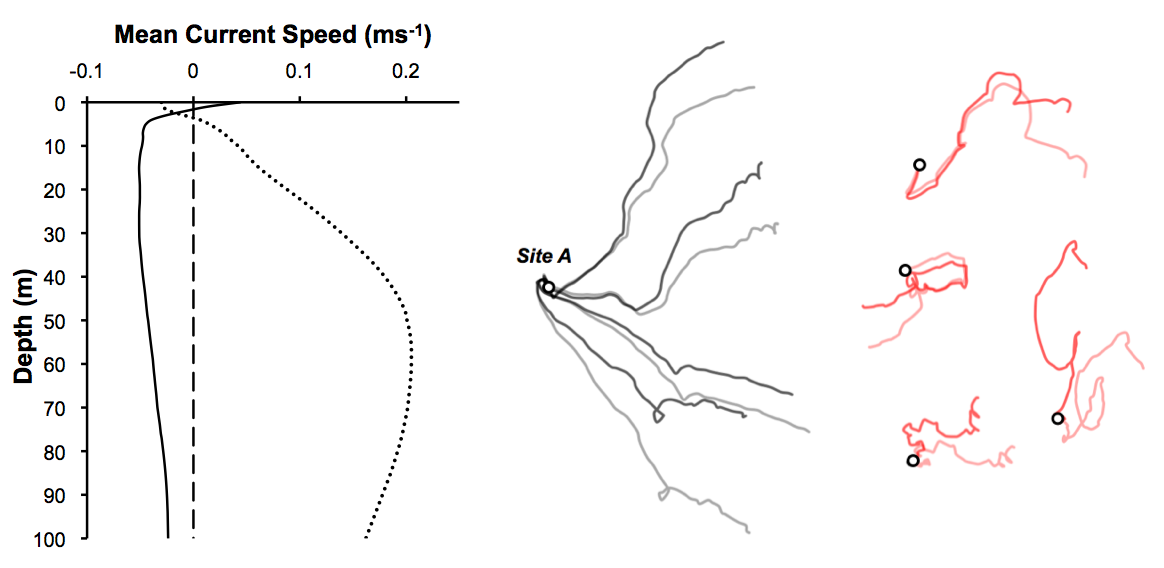

Supplement: S4 Fig — Mean current profiles over tracks of sharks released from Site A are shown on the left graph, with the zonal component as a solid black line (negative is west and positive is east) and the meridional component as a dashed black line (negative is south and positive is north). To the right are representative tracked ground paths and reconstructed motor paths of sharks released from Site A. Tracked ground paths are indicated by solid black (sham-treated) and red (anosmic) lines, while the reconstructed motor paths are indicated by gray (sham-treated) and light red (anosmic) lines. The four sham-treated examples are shown from the same release point because they could be easily combined without overlap. The four anosmic examples are separated for clarity, but the release point is Site A in all cases. (PNG) [file pone.0143758.s004.png]

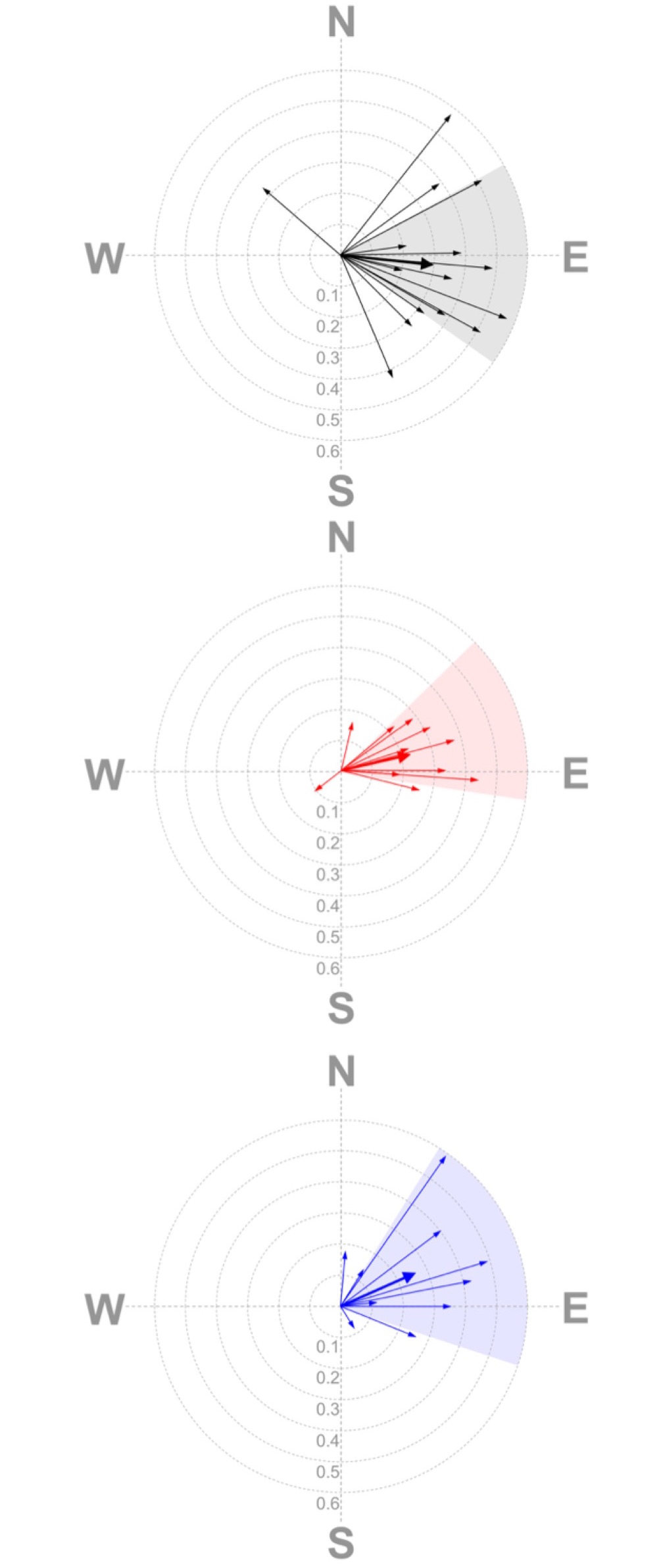

Supplement: S5 Fig — The bold arrow in each plot represents the grand MWV for the group. Shaded wedges in each plot represent the 95% confidence interval for the grand MWV. (JPG) [file pone.0143758.s005.jpg]

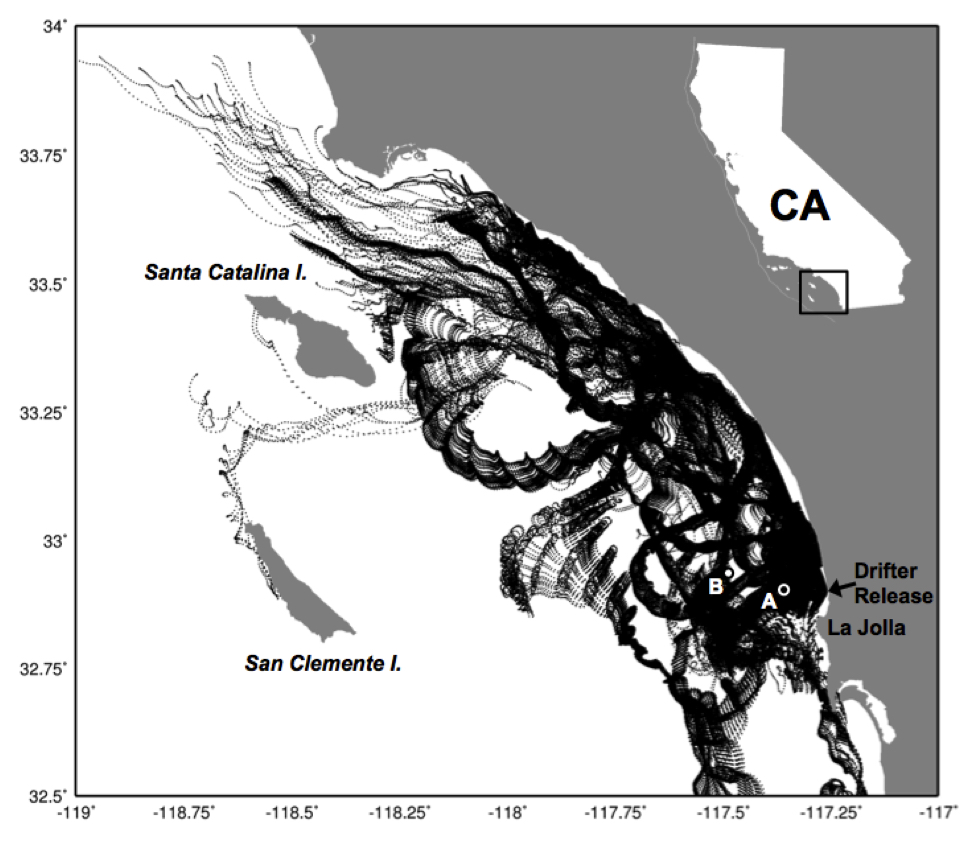

Supplement: S6 Fig — The extent of offshore and alongshore transport shown by small black dots, which indicate the simulated tracks of clusters of 100 virtual drifters released just offshore at a depth of 20 m (black arrow), directly east of release site A. Release site B is shown for reference. These drifters were released every 15 days during July–November of 2013–2014 and tracked for 17 days. (JPG) [file pone.0143758.s006.jpg]
